# Inactivation of mammalian Ero1α is catalysed by specific protein disulfide-isomerases

**DOI:** 10.1042/BJ20140234

**Published:** 2014-06-13

**Authors:** Colin Shepherd, Ojore B. V. Oka, Neil J. Bulleid

**Affiliations:** *Institute of Molecular, Cellular and Systems Biology, College of Medical Veterinary and Life Sciences, Davidson Building, University of Glasgow, Glasgow G12 8QQ, U.K.

**Keywords:** disulfide formation, endoplasmic reticulum, endoplasmic reticulum oxidase 1 (Ero1), protein disulfide isomerase (PDI), protein folding, AMS, 4-acetamido-4′-maleimidylstilbene-2,2′-disulfonic acid, ER, endoplasmic reticulum, Ero1, ER oxidase 1, ERp46, ER-resident protein 46, NEM, *N*-ethylmaleimide, PDI, protein disulfide-isomerase, PDIr, PDI-related protein

## Abstract

Disulfide formation within the endoplasmic reticulum is a complex process requiring a disulfide exchange protein such as PDI (protein disulfide-isomerase) and a mechanism to form disulfides *de novo*. In mammalian cells, the major pathway for *de novo* disulfide formation involves the enzyme Ero1α (endoplasmic reticulum oxidase 1α) which couples oxidation of thiols to the reduction of molecular oxygen to form hydrogen peroxide (H_2_O_2_). Ero1α activity is tightly regulated by a mechanism that requires the formation of regulatory disulfides. These regulatory disulfides are reduced to activate and reform to inactivate the enzyme. To investigate the mechanism of inactivation we analysed regulatory disulfide formation in the presence of various oxidants under controlled oxygen concentration. Neither molecular oxygen nor H_2_O_2_ was able to oxidize Ero1α efficiently to form the correct regulatory disulfides. However, specific members of the PDI family, such as PDI or ERp46 (endoplasmic reticulum-resident protein 46), were able to catalyse this process. Further studies showed that both active sites of PDI contribute to the formation of regulatory disulfides in Ero1α and that the PDI substrate-binding domain is crucial to allow electron transfer between the two enzymes. The results of the present study demonstrate a simple feedback mechanism of re-gulation of mammalian Ero1α involving its primary substrate.

## Abstract 2

The formation of disulfides in proteins that enter the endoplasmic reticulum is essential for their folding. We show in the present study that a key component of the machinery for disulfide formation is negatively regulated by the product of its catalytic activity.

## INTRODUCTION

The formation of disulfides within proteins entering the secretory pathway is catalysed by the PDI (protein disulfide-isomerase) family of disulfide exchange proteins [1]. These enzymes act as intermediaries, transferring disulfides from oxidases, such as Ero1 [ER (endoplasmic reticulum) oxidase 1] or peroxiredoxin IV, to substrate proteins [2]. There are two isoforms of the flavoprotein Ero1 (α and β) in mammalian cells capable of coupling the reduction of oxygen to the formation of a disulfide and hydrogen peroxide (H_2_O_2_) [3]. Both isoforms are regulated by a mechanism that involves the formation of disulfides between catalytic and non-catalytic cysteine side chains [4,5]. For the enzyme to become active these regulatory disulfides need to be broken. The regulatory disulfides then reform, thereby inactivating the enzyme and preventing any excess formation of potentially damaging H_2_O_2_.

Ero1α contains two active site cysteine pairs: a shuttle cysteine pair positioned within an extended flexible loop and an inner active site cysteine pair adjacent to the FAD cofactor [6]. The Ero1α shuttle cysteines (Cys^94^ and Cys^99^) are inactivated by the formation of two regulatory disulfides (Cys^94^–Cys^131^ and Cys^99^–Cys^104^) [4–6]. The equivalent shuttle cysteine pair in Ero1β (Cys^90^ and Cys^95^) is inactivated by the formation of a disulfide between Cys^90^ and Cys^130^. The activities of the two isoforms of Ero1 are very similar as is their mechanism of interaction with PDI so the basis for regulation is likely to be similar.

The initial reduction of the Ero1 regulatory disulfides is thought to be brought about by the action of the primary substrate PDI [6,7]. The interaction between Ero1 and PDI has been shown to be mediated through the PDI substrate-binding domain (the b′ domain) [6] with Ero1 preferentially oxidizing the second catalytic domain within PDI (the a′ domain) [7,8]. The intracellular concentration of PDI seems to determine the extent of Ero1 activation [4] giving rise to the suggestion that PDI acts as a control regulator of redox homoeostasis in the ER. If the redox conditions become too reducing then reduced PDI activates Ero1 to restore the redox poise. A more oxidizing ER would result in PDI becoming more oxidizing thereby preventing Ero1 activation.

Studies with the yeast enzyme (Ero1p) have shown a similar mechanism of regulation, although the regulatory disulfides do not involve the catalytic cysteine pairs [9]. Rather the breaking and formation of the regulatory disulfides in Ero1p have been suggested to release or restrict the movement of the flexible loop containing the shuttle cysteine pair [9]. The mechanism of reduction of the regulatory disulfide is thought to involve Pdi1p with the reformation of the inactivating disulfide occurring via an autonomous reaction that can be accelerated by oxidized Pdi1p [10]. Hence, the general mechanism of regulation of yeast and mammalian Ero1 appears to be conserved.

Although Ero1α activity is known to be regulated by intramolecular disulfide bonds, the source of the oxidizing equivalents which inactivate Ero1α activity has not yet been elucidated. In the present study we tested the hypothesis that Ero1α is regulated by one or more of three mechanisms: (i) Ero1α could generate and distribute disulfide bonds intra- or inter-molecularly via the shuttle cysteines to the regulatory cysteine pairs; (ii) H_2_O_2_ could induce sulfenylation and disulfide bond formation within the regulatory cysteines; and (iii) that members of the PDI family could directly oxidize Ero1α. The redox state of a recombinant version of Ero1α was monitored *in vitro* with a number of potential oxidants to identify the most efficient regulatory mechanism. We report that specific members of the PDI family act to rapidly oxidize and shut down Ero1α activity, providing further insight into the complicated redox balance within the ER. Further investigation of the PDI–Ero1α interaction reveals that oxidation of Ero1α by PDI is dependent on the substrate binding site within the b′ domain.

## EXPERIMENTAL

### Recombinant protein expression and purification

The expression and purification of thioredoxin, Ero1α and PDI wild-type have been described previously [5]. The PDI substrate-binding mutant (I272A, D346A, D348A) and the active site mutants ΔS1 and ΔS2 were gifts from Professor Lloyd Ruddock (University of Oulu, Oulu, Finland). The proteins were expressed and purified as described for the wild-type protein.

### Western blotting

Following electrophoresis, proteins were transferred on to nitrocellulose membranes (LI-COR Biosciences), which were then blocked for 1 h in 3% (w/v) non-fat dried skimmed milk powder in 10 mM Tris/HCl buffer (pH 7.5), containing 150 mM NaCl and 0.1% Tween 20. Blots were then incubated for 1 h with an antibody specific for Ero1α (Cell Signalling Technologies). Detection was by LI-COR IRDye fluorescent secondary antibodies typically at a 1:5000 dilution. Blots were scanned using an Odyssey Sa Imaging System (LI-COR Biosciences).

### Ero1α thioredoxin assay

Reduced thioredoxin was prepared following treatment with 10 mM DTT for 10 min at 4°C. The DTT was removed using a PD10 column (GE Healthcare) according to the manufacturer's protocol. Typically for the time course assays, 2 μM Ero1α was incubated with 100 μM reduced thioredoxin over 1800 s with samples taken at specific time points. The reactions were stopped by adding SDS sample buffer [200 mM Tris/HCl buffer (pH 6.8), containing 3% SDS, 10% glycerol, 1 mM EDTA and 0.004% Bromophenol Blue] with either 50 mM NEM (*N*-ethylmaleimide) or 20 mM AMS (4-acetamido-4′-maleimidylstilbene-2,2′-disulfonic acid) to freeze the redox state. Samples were then analysed by SDS/PAGE to visualize the Ero1α and thioredoxin redox state. For the anaerobic assays, all reagents and buffers were kept in an anaerobic chamber (Coy Laboratory Products) overnight to eliminate oxygen. The assay buffer [50 mM Tris/HCl (pH 7.5) containing 1 mM EDTA] was supplemented with 200 μM free FAD as indicated and was purged with nitrogen before incubation in the dark in the anaerobic chamber. There was no significant reduction of FAD during this incubation period.

### Ero1α re-oxidation assay

All Ero1α re-oxidation assays were carried out under anaerobic conditions in an anaerobic glove box. Reduced Ero1α was prepared following incubation with 10 mM DTT for 1 min before applying to a micro Bio-Spin 6 chromatography column (Bio-Rad Laboratories) which was pre-equilibrated in anaerobic buffer [50 mM Tris/HCl (pH 7.5) and 1 mM EDTA] to remove DTT. For the oxygen titration time course assay, the reduced Ero1 samples were incubated with combinations of anaerobic and air-saturated buffer (approximately 250 μM oxygen) containing 50 mM Tris/HCl (pH 7.5) and 1 mM EDTA to a final oxygen concentration of 50 μM and 225 μM. Following incubation, the reactions were stopped at various times (0, 10, 30, 60 and 300 s) by adding SDS sample buffer [200 mM Tris/HCl buffer (pH 6.8), 3% SDS, 10% glycerol, 1 mM EDTA and 0.004% Bromophenol Blue] containing 25 mM NEM. Samples were analysed by SDS/PAGE under non-reducing conditions and Ero1α visualized by silver staining.

For the oxidoreductase time course assays, PDI, ERp46 (ER-resident protein 46) or PDIr (PDI-related protein) were oxidized by incubation with 20 mM GSSG for 15 min before applying to Micro Bio-Spin columns to remove GSSG. The oxidized proteins were incubated with 2 μM reduced Ero1α in an anaerobic buffer [50 mM Tris/HCl (pH 7.5) and 1 mM EDTA]. Samples were taken at time points 0, 10, 30, 60 and 300 s and immediately alkylated in SDS sample buffer containing 25 mM NEM. Samples were analysed by SDS/PAGE and visualized by Western blotting.

For the H_2_O_2_, GSSG and thioredoxin assays each reagent was incubated at the indicated concentrations with 2 μM reduced Ero1α. Samples were taken at 0, 10, 30, 60 and 300 s, followed by alkylation in SDS sample buffer containing 25 mM NEM. Samples were analysed by SDS/PAGE and visualized by silver staining. Densitometry was then performed on scanned gels using unmodified output images and ImageJ (NIH).

## RESULTS

### Formation of disulfides in Ero1α is prevented under anaerobic conditions

**Figure 1:**
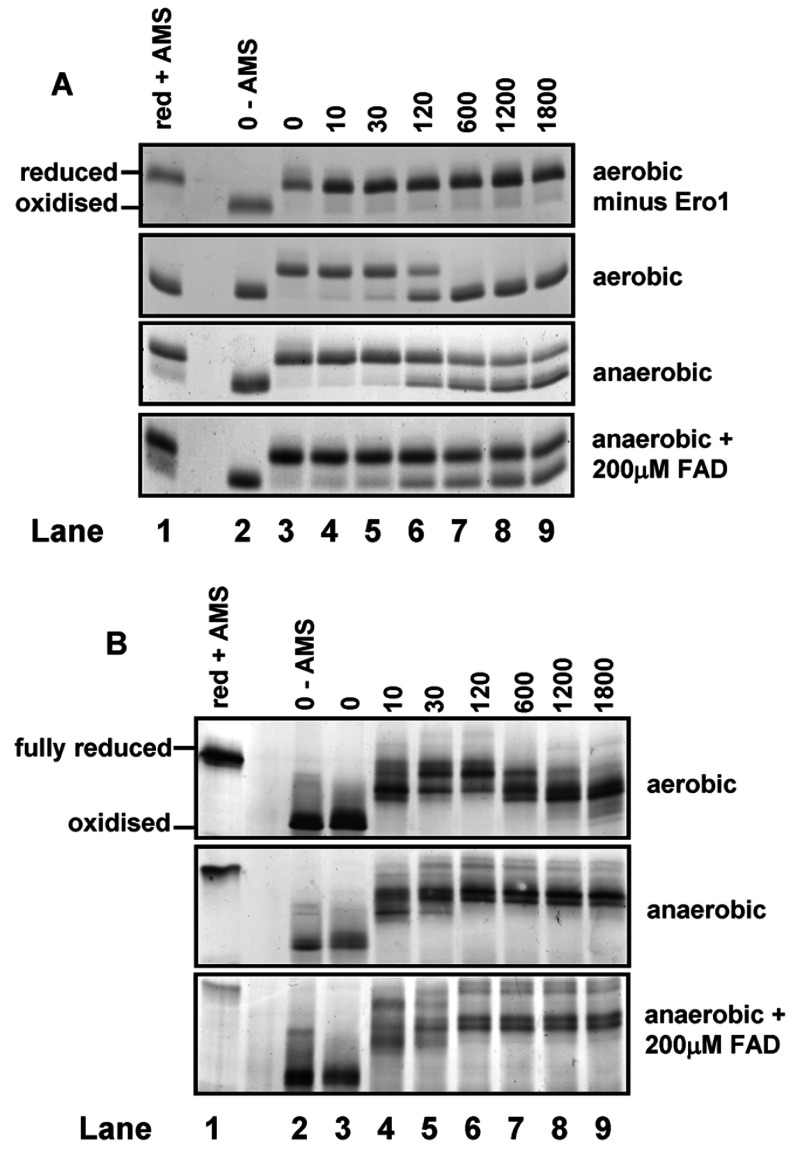
Oxygen is essential for the oxidation of thioredoxin by Ero1α Reduced thioredoxin (100 μM) was incubated with Ero1α (2 μM) and at the indicated time points (annotated in seconds) samples were alkylated with AMS to freeze the redox state of both enzymes. Samples were then analysed by non-reducing SDS/PAGE and Coomassie Blue stained to visualize thioredoxin (**A**) or silver stained to visualize Ero1α (**B**). Reduced and oxidized controls are included for comparison. The assay was carried out under aerobic or anaerobic conditions and in the presence or absence of Ero1α as indicated. FAD (200 μM) was also added to the reactions carried out under anaerobic conditions as indicated. The results are representative of three separate experiments.

To establish an assay for the re-oxidation of the regulatory disulfides in Ero1α, we incubated Ero1α with a model substrate, thioredoxin under both aerobic and anaerobic conditions. At various time points after initiation of the reaction, samples were alkylated with AMS, which adds ~510 Da per modified thiol, before separation by SDS/PAGE (Figure 1). The oxidized forms of thioredoxin and Ero1α migrate comparatively quickly through the gel due to their decreased hydrodynamic radius and mass compared with the reduced forms which react with AMS. In the absence of Ero1α and under aerobic conditions, thioredoxin remains in the reduced slow-migrating form (Figure 1A, top panel) throughout the time course. When Ero1α was included under aerobic conditions, the redox state of thioredoxin shifts completely from the slower-migrating reduced form, witnessed at 0 and 10 s, to the oxidized form between 120 and 600 s (Figure 1A, upper middle panel). When the assay was carried out under anaerobic conditions, the oxidation of thioredoxin was dramatically inhibited (Figure 1A, lower middle panel), confirming the oxygen dependence of the catalytic activity of Ero1α. Furthermore, we assessed the ability of exogenous FAD to act as an alternative terminal electron acceptor for Ero1α. Our findings show that the inclusion of exogenous FAD in this assay does not restore Ero1α activity, as oxidation of thioredoxin was not increased in the presence of FAD (Figure 1A, bottom panel).

We and others have shown that the redox status of Ero1α changes during its reaction with thioredoxin, specifically the regulatory disulfides are first reduced during substrate oxidation and then are themselves oxidized [4,5]. This transition in redox status can be followed by changes in the mobility of the protein when separated without prior reduction and these changes have been shown to be due to the reduction of the regulatory disulfides by mutation of individual cysteine residues [5]. The redox transitions can also be followed by AMS modification during the reaction of Ero1α with thioredoxin under aerobic conditions (Figure 1B, top panel). An initially oxidized Ero1α quickly becomes reduced, after 10–120 s, in the presence of reduced thioredoxin. This redox state shift coincides with the oxidation of thioredoxin (Figure 1A). The Ero1α redox state then shifts towards a more oxidized state between 600–1800 s, after the oxidation of thioredoxin is complete. It is of interest to note that Ero1α does not return to its original redox state within the time course of the assay indicating that some disulfides reform relatively slowly. Under anaerobic conditions, the redox state of Ero1α again displays a shift from oxidized to a reduced form between 0–120 s; however, this form of Ero1α does not shift towards the more oxidized form at 600–1800 s (Figure 1B, middle panel). Hence once the disulfides are reduced by thioredoxin, they do not reform if no oxygen is present in the reaction buffer. In addition, in the presence of exogenous FAD the redox state of Ero1α follows a similar pattern to that in its absence, confirming that non-enzyme bound FAD cannot act as an oxidant to regenerate disulfides (Figure 1B, bottom panel). These results clearly demonstrate that, in contrast with the yeast enzyme [11,12], Ero1α cannot utilize FAD as an alternative electron acceptor.

### The correct regulatory disulfides within Ero1α are re-oxidized slowly in the presence of oxygen or H_2_O_2_

**Figure 2:**
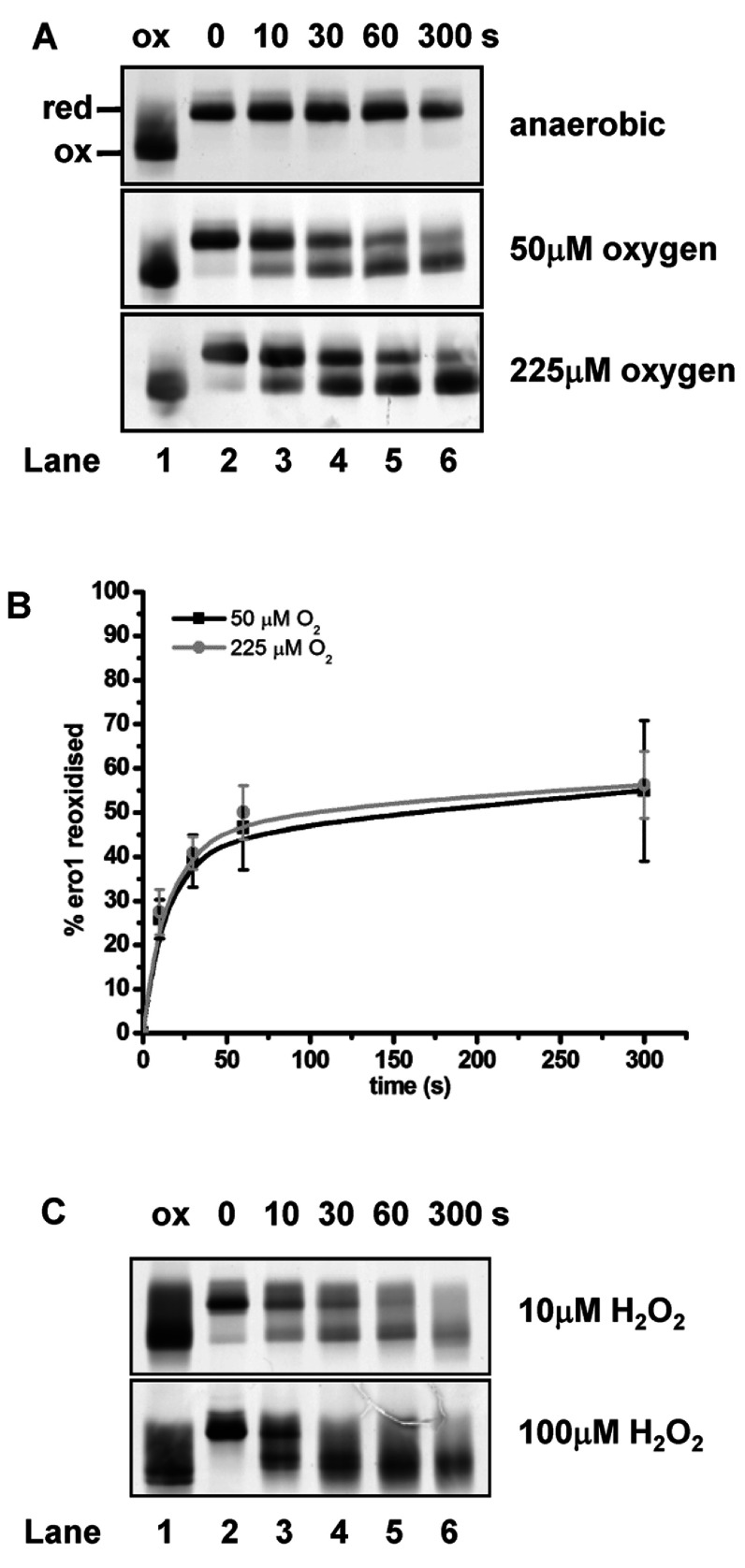
Autonomous oxidation of Ero1α by oxygen and H_2_O_2_ Reduced Ero1α (2 μM) was incubated either under anaerobic conditions or with increasing concentrations of oxygenated buffer (**A**) or H_2_O_2_ (**C**). The redox state of Ero1α was frozen by alkylating with NEM at the given time points (annotated in seconds), before analysing the samples by non-reducing SDS/PAGE and silver staining. The positions of the fully reduced (red) and oxidized (ox) forms are as indicated. (**B**) The fraction of Ero1α re-oxidized in the presence of oxygen for three independent experiments was quantified and the mean value calculated and plotted against the time of incubation (error bars represent S.D.).

Having established that disulfides within Ero1α once reduced do not reform under anaerobic conditions, we wanted to determine whether oxygen or H_2_O_2_ could bring about the reformation of these disulfides. We developed an assay whereby Ero1α could be stabilized in the reduced state under anaerobic conditions before monitoring its re-oxidation after the re-addition of oxygen to the system. Ero1α was reduced with DTT under anaerobic conditions before removal of the reducing agent. After the addition of oxygen, the alkylating agent NEM was added at various time points to freeze the redox state and samples were analysed by SDS/PAGE carried out under non-reducing conditions. There is a distinct difference in the mobility of reduced and oxidized Ero1α when separated under non-reducing conditions due to the formation of the long-range regulatory disulfides (Figure 2A, lanes 1 and 2) [5]. Ero1α remained in the reduced state under anaerobic conditions throughout the 300 s time course (Figure 2A, top panel). Introducing 50 μM oxygen into the buffer with reduced Ero1α resulted in the partial oxidation of Ero1α over 300 s (Figure 2A, middle panel). Increasing the concentration of oxygen in the buffer to 225 μM did not further increase the rate or amount of Ero1α re-oxidation (Figure 2A, bottom panel). The partial oxidation of Ero1α by oxygen indicates that disulfide exchange within or between Ero1α molecules can occur albeit inefficiently to re-oxidize the regulatory disulfides.

H_2_O_2_ is produced by Ero1α as a consequence of disulfide bond formation. Hence we determined whether H_2_O_2_ may induce sulfenylation of cysteine thiols within Ero1α leading to regulatory disulfide formation. The redox state of Ero1α was followed over 300 s following the addition of either 10 or 100 μM H_2_O_2_. In the presence of 10 μM H_2_O_2_, Ero1α shifts from the reduced to the oxidized state (Figure 2C, upper panel). Oxidation begins almost immediately and the proportion of oxidized Ero1α increases until 300 s. The inclusion of 100 μM H_2_O_2_ results in increased Ero1α oxidation; however, the bands corresponding to the oxidized form of the enzyme are diffuse and difficult to quantify, suggesting several different disulfide linked products (Figure 2C, lower panel). These results demonstrate that whereas H_2_O_2_ may provide a means of Ero1α oxidation, an ensemble of forms are produced with different combinations of disulfides. Hence it is unlikely that H_2_O_2_ itself leads to the formation of the regulatory disulfides within Ero1α.

### Ero1α is oxidized rapidly by specific ER oxidoreductases

**Figure 3:**
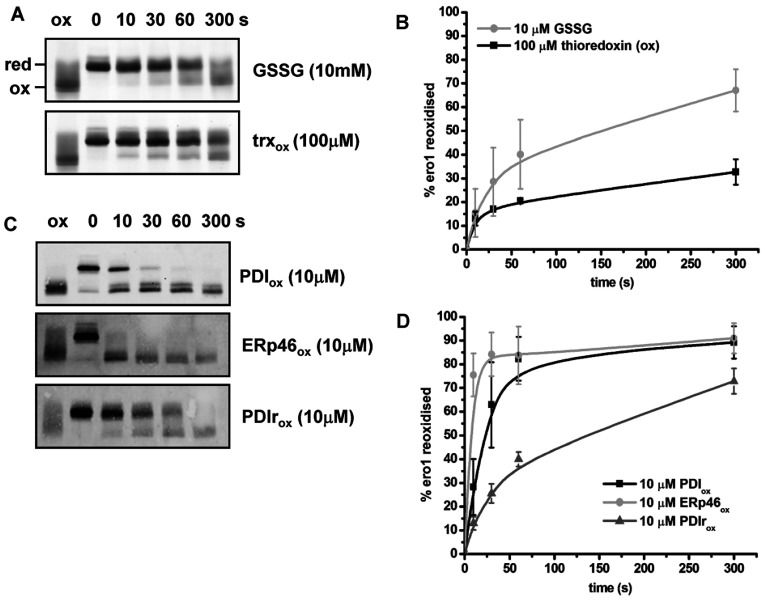
Ero1α is oxidized rapidly and efficiently by specific ER oxidoreductases Reduced Ero1α (2 μM) was incubated under anaerobic conditions with (**A**) either GSSG (10 mM) or oxidized thioredoxin (100 μM) or (**C**) oxidized PDI, ERp46 or PDIr all at 10 μM. After the time points indicated (seconds) the redox state of Ero1α was frozen with NEM and samples analysed by non-reducing SDS/PAGE and silver stain (**A**) or Western blot with an antibody against Ero1α (**C**). ox, oxidized; red, reduced. (**B** and **D**) The fraction of Ero1α re-oxidized for three independent experiments was quantified and the mean value calculated and plotted against the time of incubation (error bars represent S.D.).

The glutathione buffer within the ER along with the PDI family of enzymes could facilitate the formation of the regulatory disulfide bonds within reduced Ero1α. To determine whether individually these factors could reform the Ero1α regulatory disulfides we incubated reduced Ero1α, under anaerobic conditions, with GSSG or various oxidized oxidoreductases. In the presence of 10 mM GSSG and 2 μM Ero1α, the regulatory disulfides were formed relatively slowly and to a limited extent after 300 s (Figure 3A, upper panel). GSSG itself is therefore unlikely to regulate Ero1α activity. In addition, 100 μM oxidized thioredoxin exchanged its disulfide with Ero1α only to a limited extent (Figure 3A, lower panel) suggesting that this enzyme inefficiently oxidizes the regulatory disulfides in the absence of oxygen. Reduced Ero1α was also incubated with various ER oxidoreductases, including PDI, ERp46 and PDIr (Figure 3C). These proteins were chosen due to the availability of purified recombinant protein and their known role as good (PDI) or poor (ERp46) substrates for Ero1α [13]. PDI and ERp46 were able to oxidize Ero1α regulatory disulfides quickly and efficiently at low molar ratios (2 μM Ero1α and 10 μM oxidoreductase) within 10 s and 30 s respectively. PDIr was also able to re-oxidize Ero1α, but with slower kinetics (Figure 3C, bottom panel). These results demonstrate that oxidized PDI family members are able to exchange disulfides with Ero1α in the absence of oxygen resulting in the direct reformation of the regulatory disulfide. The efficiency of the exchange reaction is likely to be dependent on the reduction potential of the disulfide within the thioredoxin domains as oxidized thioredoxin with a relatively low reduction potential was unable to efficiently exchange its disulfide with Ero1α.

### Oxidation of Ero1α by the individual thioredoxin domains of PDI is influenced by the substrate-binding domain of PDI

**Figure 4:**
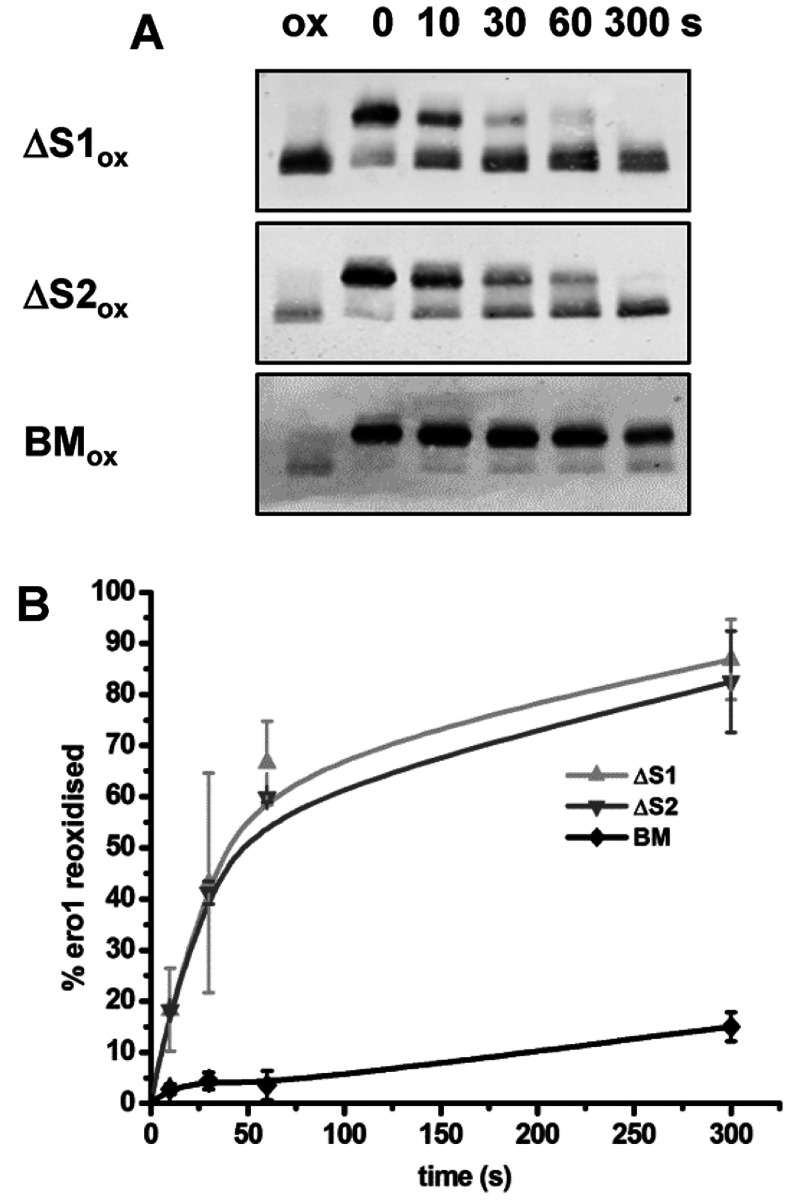
Both PDI active sites contribute to Ero1α re-oxidation and inactivation, as does the PDI b′-binding domain Reduced Ero1α (2 μM) was incubated under anaerobic conditions with the oxidized (ox) PDI mutants ΔS1 or ΔS2 or the PDI-binding mutant (BM) all at 10 μM. (**A**) After the time points indicated (seconds) the redox state of Ero1α was frozen with NEM and samples analysed by non-reducing SDS/PAGE and Western blotting with an antibody against Ero1α. (**B**) The fraction of Ero1α re-oxidized for three independent experiments was quantified and the mean value calculated and plotted against the time of incubation (error bars represent S.D.).

Previous studies have shown that reduced PDI interacts with active Ero1α during its oxidation via the PDI substrate-binding domain [6,8,14] and that Ero1α preferentially oxidizes the PDI a′ domain active site [7,8]. To investigate whether a similar interaction between reduced Ero1α and oxidized PDI is required for the reformation of the regulatory disulfides we determined the ability of three PDI mutants to exchange disulfides with Ero1α. These were: ΔS1, which has the a′ domain active site mutated to AGHA; ΔS2, which has the a domain active site mutated to AGHA; and BM, which has three point mutations (I272A, D346A and D348A) within the b′ domain that prevent substrate binding [15]. Each mutant was assessed for its ability to exchange disulfides with reduced Ero1α under anaerobic conditions (Figure 4). Using the PDI active site AGHA mutants ensure that any disulfide exchange between PDI and Ero1α can only be via the remaining intact active site. The ΔS1 mutant was able to oxidize Ero1α at a similar rate to the ΔS2 mutant (Figure 4A, top and middle panels). Strikingly, the PDI substrate-binding mutant (BM) was not able to efficiently exchange its disulfides with Ero1α (Figure 4A, bottom panel). These results suggest that there is no specificity in the ability of the two active site domains of PDI to exchange disulfides with Ero1α, although the substrate-binding domain is critical to allow the exchange to occur.

## DISCUSSION

**Figure 5:**
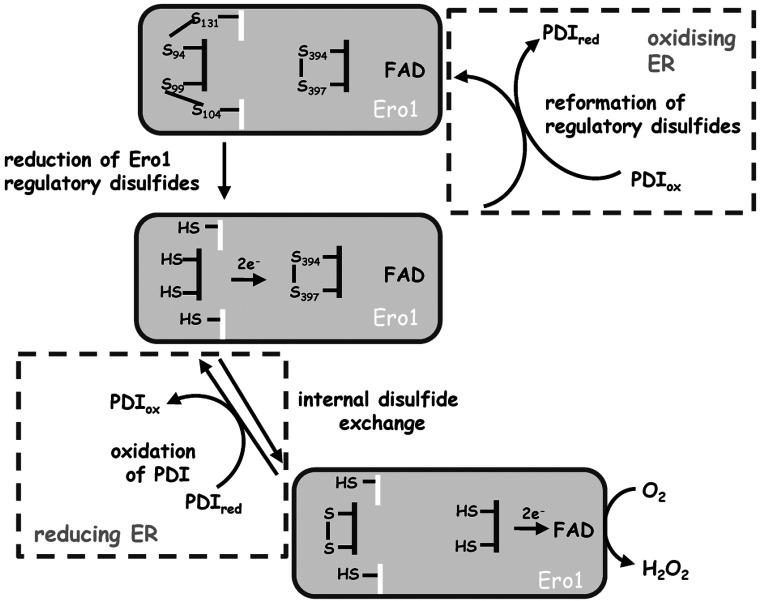
Regulation of the Ero1α redox cycle by PDI oxidoreductases The activation of Ero1 occurs via a two-step reaction mechanism, namely the reduction of its regulatory disulfide by PDI, followed by an intramolecular disulfide exchange with the inner active site disulfide proximal to the isoalloxazine ring of FAD. Under more reducing ER conditions Ero1 is activated to oxidize PDI. The oxidation of PDI is coupled to the reduction of molecular oxygen to produce H_2_O_2_. When ER conditions are more oxidizing, oxidized PDI oxidoreductases can catalyse the reformation of the regulatory disulfide bond in Ero1, leading to its inactivation. ox, oxidized; red, reduced.

The activity of Ero1α in mammalian cells is tightly regulated to prevent excessive oxidative stress due to the production of H_2_O_2_. This regulation comes principally from two intramolecular disulfides that, when formed, inhibit Ero1α activity [4,5]. The Ero1α substrate, PDI, is able to reduce the regulatory disulfides within Ero1α in order to activate the enzyme and in doing so regulates the formation of its own active site disulfide [16]. Until recently, it was unknown how these regulatory disulfides reform to inactivate the enzyme or what contribution to this process is made by autonomous oxidation or by enzymatic catalysis. It has been demonstrated that the regulatory disulfides in the yeast enzyme can be reformed by molecular oxygen and that this process can be accelerated by Pdip [10], but it was still an open question as to whether a similar mechanism exists within mammalian cells and whether there is any specificity in the oxidoreductases capable of catalysing this process. The conclusion from the present study is that whereas the regulatory disulfides in Ero1α can be reformed by oxygen in the absence of an enzymatic catalyst, the rate of reformation is dramatically enhanced in the presence of specific oxidized PDI family members even in the absence of oxygen. The ability of PDI to catalyse the activation and inactivation of its own catalyst provides an elegant mechanism to ensure PDI can fulfil its role as a disulfide exchange protein both introducing and removing disulfides from substrate proteins (see Figure 5). It has been shown that the presence of constitutively active Ero1α in cells leads to an increase in the amount of oxidized PDI [4], which would compromise the ability of PDI to reduce non-native disulfides in substrates.

We also show that Ero1α does not use FAD as an alternate electron acceptor to drive disulfide formation. These results contrast with those showing that FAD can act as an electron acceptor for Ero1p both in an *in vitro* assay [11,12,17] and *in vivo* [18]. The reasons for this difference in the ability of yeast and mammalian Ero1 to utilize exogenous FAD is unclear, but highlights the subtle differences between these two enzymes which also includes the lack of conservation of the regulatory disulfides. However, the regulatory disulfides in both enzymes can be reformed in the presence of oxygen alone which suggests that an internal disulfide rearrangement within the enzyme can result in disulfides being shuttled from the cysteine pair proximal to the isoalloxazine ring of FAD to the shuttle cysteines and finally to the regulatory cysteine pair. The fact that PDI can catalyse the formation of the regulatory disulfides in the absence of oxygen suggests that this disulfide exchange occurs directly with either the active site cysteine pair or directly with the regulatory cysteines.

As H_2_O_2_ is produced by Ero1α as a direct consequence of its activity [17] it was important to determine its ability to reform the regulatory disulfides. Unlike oxygen, H_2_O_2_ could directly oxidize the thiol groups to form sulfenylated cysteine which would quickly be resolved by adjacent thiols to form a disulfide. Our results suggest that, whereas H_2_O_2_ was able to oxidize reduced thiols in Ero1α, there was a lack of specificity with several differently disulfide bonded species being formed. It is still an open question what the concentration of H_2_O_2_ is in the ER. Although Ero1α may well be a source of this molecule there are several peroxidases, such as peroxiredoxin IV [19] and glutathione peroxidase 7 and 8 [20], that are highly active and would be able to rapidly remove any H_2_O_2_ produced by Ero1α. Hence the lack of specificity in directing disulfide formation and the potentially low concentrations make it unlikely that H_2_O_2_ is involved in the reformation of the regulatory disulfides in the ER.

Previous data have suggested that Ero1α binds to PDI via a protruding β-hairpin structure which interacts specifically with a binding pocket within the PDI b′ domain [6]. In the present study we find that disruption of the binding pocket by mutating three key residues has a similar effect on the ability of PDI to exchange disulfides with Ero1α to reform the regulatory disulfides. Although the substrate-binding function of PDI is essential for its ability to interact with Ero1α, ERp46 is able to efficiently exchange disulfides with Ero1α despite the absence of a similar non-catalytic substrate-binding domain. Previous work has shown that ERp46 is a substrate for Ero1α, albeit at a much reduce rate compared with PDI [13]. Given the efficiency of inactivation of Ero1α by oxidized ERp46 it is unlikely that the slow oxidation of ERp46 is due to a lack of physical interaction between these two proteins. Hence the catalytic domains of ERp46 must be able to efficiently interact with Ero1α to reform the regulatory disulfides circumventing the requirement for a non-catalytic substrate-binding domain. In contrast it may well be the case that the efficient oxidation of PDI family members by Ero1α requires the presence of a non-catalytic substrate-binding site, a possibility that is supported by the efficient oxidation of ERp57 when its b′ domain is replaced with the same domain from PDI [6].

Taken together, the data presented in this study of Ero1α regulation reveal that, whereas molecular oxygen and H_2_O_2_ can induce disulfide formation within Ero1α, the enzymatic catalysis of disulfide exchange by specific PDI family members is the most efficient mechanism of activity regulation. The consequence of this tight regulation and the presence of efficient ER peroxidases is that excessive H_2_O_2_ is unlikely to accumulate within the ER of mammalian cells following disulfide formation in mammalian cells.
